# Tin–Carbon Dual Buffer Layer to Suppress Lithium Dendrite Growth in All-Solid-State Batteries

**DOI:** 10.1021/acsnano.4c16271

**Published:** 2025-04-29

**Authors:** Venkata Sai Avvaru, Tofunmi Ogunfunmi, Seonghun Jeong, Mouhamad Said Diallo, John Watt, Mary C. Scott, Haegyeom Kim

**Affiliations:** † Materials Sciences Division, Lawrence Berkeley National Laboratory, Berkeley, California 94720, United States; ‡ Department of Materials Science and Engineering, University of California, Berkeley, California 94720, United States; § Molecular Foundry Division, Lawrence Berkeley National Laboratory, Berkeley, California 94720, United States; ∥ Center for Integrated Nanotechnologies, Los Alamos National Laboratory, Los Alamos, New Mexico 87545, United States

**Keywords:** buffer layer, lithium metal, dendrite, solid-state battery, carbon

## Abstract

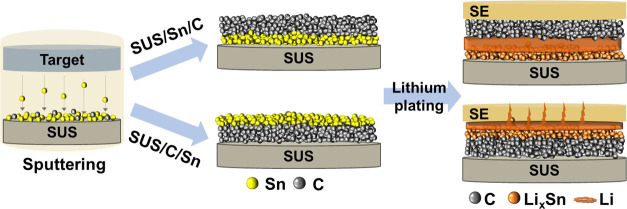

All-solid-state lithium–metal batteries hold great promise because of their high energy density stemming from using an energy-dense lithium–metal anode. However, mitigating the dendritic lithium–metal growth, originating from heterogeneous lithium–metal deposition, is a priority to suppress short-circuit and extend cycle life. This study employs direct current (DC) magnetron sputter coating to deposit tin (Sn) and carbon (C) on a stainless steel (SUS) current collector to achieve uniform lithium–metal plating and improve cycling performance. In particular, we evaluated and compared two dual buffer layer designs, consisting of Sn and C: (1) a thin C layer is deposited on the Sn metal layer (SUS/Sn/C), and (2) the Sn metal layer is deposited on the thin C layer (SUS/C/Sn). This study demonstrated that the SUS/Sn/C buffer layer is more effective in suppressing lithium dendrite growth and improving cycling stability than the SUS/C/Sn buffer layer. The SUS/Sn/C buffer layer shows stable Li-plating/stripping cycling over 450 cycles without noticeable short-circuit. *Ex situ* and *in situ* characterization confirm the role of the SUS/Sn/C dual buffer layer: (i) the Sn metals result in a uniform lithium–metal deposition on the current collector and (ii) the carbon layer acts as a physical barrier to suppress the lithium dendrite growth toward the solid electrolyte because of its lithiophobic nature.

Solid-state batteries (SSBs) have attracted significant interest from both academia and industry as next-generation high-energy rechargeable batteries. SSBs employ solid-state inorganic compounds as lithium (Li)-conducting electrolytes instead of flammable liquid organic electrolytes, improving the safety of rechargeable batteries.^1−3^ The higher thermal stability of solid electrolytes increases the resistance to thermal runaway compared to that of Li-ion batteries utilizing liquid electrolytes.^2^ This improved safety enables the use of a Li-metal anode, which exhibits high theoretical specific capacity (3860 mAh g^–1^) and low potential (−3.04 V vs the standard hydrogen electrode (SHE)).^4−7^

An anode-less configuration (or Li reservoir-free system) that does not contain an excess Li-metal anode but utilizes Li from a cathode to deposit on a current collector during charging of the full cell is an ideal system to maximize the specific energy (Wh kg^–1^) of the SSBs.^8,9^ In addition, the anode-less concept can simplify the SSB manufacturing process by removing the Li-metal anode manufacturing and integration steps, which can reduce production cost.^9,10^ However, the anode-less system is prone to create inhomogeneous Li-metal plating instead of the desired uniform Li deposition, leading to a short-circuit, in particular at low stacking pressures and high current rates.^11−13^

Several buffer layers (BLs) have been proposed to create uniform Li-metal plating during charging of full-cells (or discharging in Li-metal half-cells) and improve the interfacial stability between the Li-metal anode and solid electrolytes. These BLs include carbon,^14,15^ metal particles,^16−23^ carbon–metal composites,^24−30^ and other inorganic materials.^31−33^ For example, Lee et al. developed a silver–carbon (Ag–C) composite BL and demonstrated that the thin Ag–C BL effectively regulates Li deposition, which improves the cycling stability to up to 1000 cycles with a relatively high cathode loading of 6.8 mAh cm^–2^ at 60 °C.^24^ Wu et al. found that Ag nanoparticles play a key role in Ag–C composite BLs, where Ag nanoparticles drive Li-metal plating between the Ag–C BL and pre-existing Li metal, rather than at the interface between the BL and solid electrolyte.^25^ The crystallinity and microstructure of carbon BLs has also been found to determine the uniformity of Li-metal plating and the position of Li-metal deposition (at the current collector/carbon BL interface vs at the carbon BL/solid-electrolyte interface).^14,15^ Recently, various metal nanoparticles and films have been investigated as BLs and current collectors to homogenize Li-metal plating.^13,16,17,19,20,34^ Although it is difficult to exclude external parameters such as the particle size, film thickness, and stacking pressure that could affect the electrochemical Li plating/stripping performance, in general, metals that can alloy with Li up to high Li concentrations and that have good affinity with Li metal exhibit stable Li plating/stripping cycles.^16−18,20,26^ For example, Zn metal exhibits inferior cycling performance compared to Au, Ag, Mg, and Sn.^17,20^ Recently, Xie and colleagues claimed that the ability to absorb more Li makes the current at the anode more controlled by the dispersed metal nanoparticles and that uniform distribution of metal nanoparticles would help homogenize Li-metal plating.^35^ However, the role of the carbon in the composite BL is not yet fully understood, although a few studies have demonstrated that the metal–carbon composite or dual-layered BL delivers improved cycling performance compared to metal-only or carbon-only BLs.^26,30^

In this study, we developed and compared two distinct designs of dual BLs, consisting of Sn and carbon to understand the role of the carbon layer in preventing Li dendrite growth.^1^ The Sn layer was deposited on a stainless steel (SUS) current collector, and the carbon layer was deposited on the Sn layer (hereafter, SUS/Sn/C).^2^ The carbon layer was deposited on a SUS current collector, and the Sn layer was deposited on top of the carbon layer (hereafter, SUS/C/Sn). Sn only BL and SUS foil without BL were also evaluated as control groups. While Sn metal BLs have recently been extensively investigated by several groups,^17,19,36,37^ they focused on evaluating Sn-based BLs in Li excess systems (combining Li–Sn alloy and Li metal). In contrast, this study investigated the Sn-based BL in anode-less configuration (or Li reservoir-free system). In addition, to the best of our knowledge, this is the first report, investigating the role of carbon position in two different SUS/Sn/C and SUS/C/Sn BL configurations. Through electrochemical evaluations at a current density of 1 mA cm^–2^ at 50 °C, we confirmed that the SUS/Sn/C BL outperformed the SUS/C/Sn BL and Sn-only BL. We demonstrated that the carbon layer on top of the Sn layer extends Li plating/stripping cycles without short-circuiting. *Ex situ* and *in situ* optical microscopy analysis demonstrated that Li-metal plating occurred at the location where Sn metal exists (between the carbon layer and SUS foil current collector) in the SUS/Sn/C BL. In contrast, Li metal deposition occurs at the interface between the BL and the solid electrolyte in the SUS/C/Sn BL, which cannot avoid direct contact between Li metal and the solid electrolyte. In addition, in the SUS/Sn/C dual buffer layer, the carbon layer serves as a physical protective layer to suppress Li dendrite growth toward a solid electrolyte due to its lithiophobic properties.

## Results

### Dual Buffer Layer Designs

**Figure 1:**
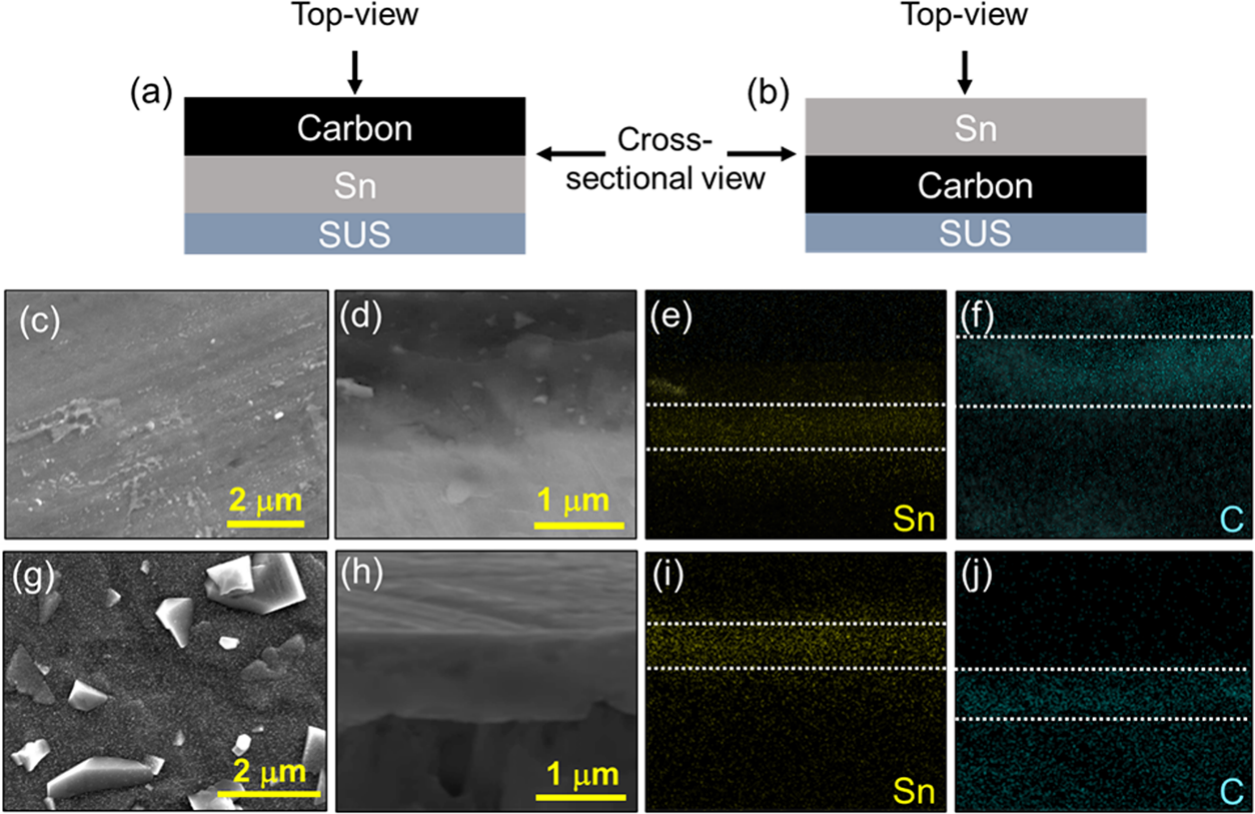
Schematic representation of (a) carbon deposited on Sn-nanoparticle layer (SUS/Sn/C) and (b) carbon deposited between the Sn and SUS foil (SUS/C/Sn). SEM images of SUS/Sn/C showing (c) top-view and (d) cross-sectional view and corresponding EDS mapping of (e) Sn and (f) C layers. SEM image of SUS/C/Sn showing (g) top view and (h) cross-sectional view and corresponding EDS mapping of (i) Sn and (j) C layers.

Several groups have demonstrated that carbon–metal composite BLs outperform metal-only or carbon-only BLs.^24−26,30^ However, the role of carbon layers and how the position of the carbon layer affects the Li plating (discharging)/stripping (charging) performance remain elusive. To close this knowledge gap, we designed and evaluated two distinct BL configurations: carbon layer is deposited (i) on top of the Sn metal layer (denoted as SUS/Sn/C) and (ii) between the Sn metal layer and SUS foil (denoted as SUS/C/Sn), as shown in the schematic in Figure 1a–b. The sputtering was conducted inside an Ar-filled glovebox, thereby preventing any potential oxidation of Sn metals due to air exposure during sputtering and sample transfer. X-ray diffraction (XRD) analysis for the Sn metal-deposited SUS foils also confirmed crystalline Sn metal signals along with the SUS foil substrate as shown in Figure S1a. In this study, we selected Sn metals as a potential BL component for several reasons. (i) Sn is capable of absorbing a large amount of Li ions, which keeps the electrochemical potential positive and may control the current uniformly at the anode before Li-metal plating occurs.^35^ (ii) Previous studies demonstrated Li–Sn alloys as working BLs in Li-rich anode systems.^17,19,36,37^ (iii) Sn metals are mechanically soft, ensuring good contact with the current collector and solid electrolyte.

Figure 1c displays a top-view scanning electron microscopy (SEM) image of the carbon deposition over the Sn metals, which demonstrates that the carbon layer is uniformly coated over the Sn metal layer because no Sn metal particles are observed in contrast to the Sn sputtered sample (Figure S1b,c). The Sn sputtered SUS (Figure S1b,c) shows a bimodal particle size distribution of <200 nm and ∼1 μm. We controlled the sputtering conditions (such as deposition power and time), but we could not create a uniform particle size distribution of Sn metals. While the sputtered Sn shows bimodal size distribution in our system, it is noteworthy to mention that all the SUS foil surface is covered by Sn particles. Figure 1d–f present an SEM image of the cross-sectional view of the SUS/Sn/C BL and corresponding EDS mapping. The thickness of the SUS/Sn/C BL was estimated from the cross-sectional images, which corresponds to ∼1.2 μm in total, where Sn is ∼500 nm and C is ∼700 nm. In contrast, the SEM image in Figure 1g clearly shows the bimodal distribution of Sn nanoparticles on the surface of the SUS/C/Sn BL. The morphology of Sn particles remains unchanged even after depositing over the carbon layer except that the particle size slightly increased compared to Sn only sputtered sample shown in Figure S1b,c. The thickness estimated from the SEM image and corresponding EDS mapping (Figure 1h–j) of the cross-sectional view of SUS/C/Sn BL is ∼1 μm. EDS elemental mapping shows a dual layer of carbon (bottom) and Sn nanoparticles (top).

**Figure 2:**
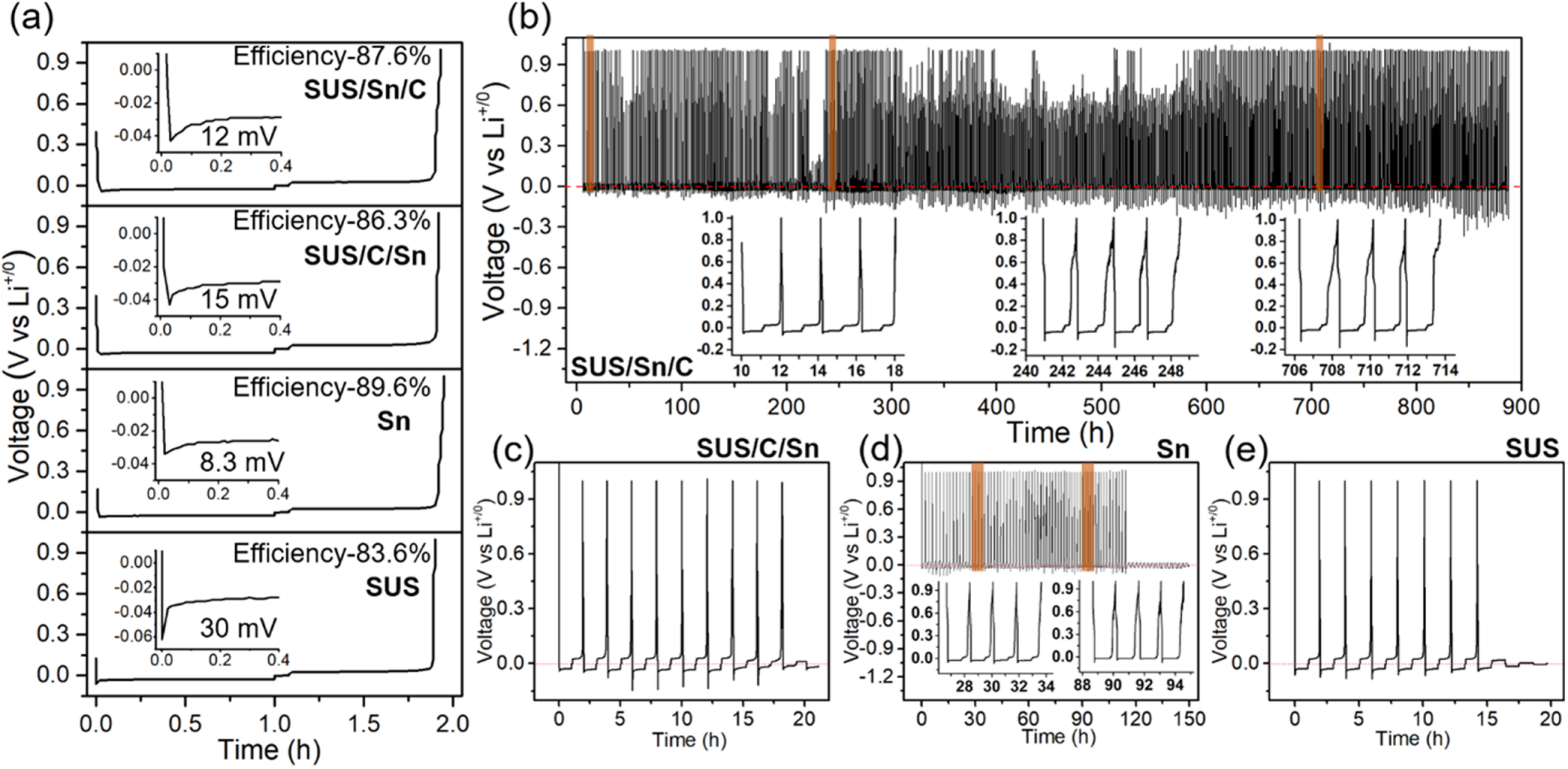
(a) First cycle voltage curves during discharge/charge cycle of SUS/Sn/C BL, SUS/C/Sn BL, Sn only BL, and bare SUS foil, respectively. The inset shows the first 12 min of discharge curves. Galvanostatic cycling test results of (b) SUS/Sn/C BL, (c) SUS/C/Sn BL, (d) Sn only BL, (e) bare SUS foil at a current density of 1 mA cm^–2^.

To understand the role of the position of the carbon layer in the BL, asymmetric cells were assembled with an argyrodite solid electrolyte (Li_6_PS_5_Cl) and a Li-metal counter electrode. The asymmetric cells were cycled at a current density of 1 mA cm^–2^ until the areal capacity reached 1 mAh cm^–2^ or the voltage reached the cutoff of −1.0 V (vs Li/Li^+^) during plating (discharging) and 1.0 V (vs Li/Li^+^) during stripping (charging) at 50 °C. In this study, we selected a slightly elevated temperature, 50 °C, for cycling to mitigate potential Li dendrite growth from the counter electrode that does not have BLs. Figure 2a shows the first Li-plating (discharging)/stripping (charging) profiles of SUS/Sn/C and SUS/C/Sn BLs, compared to bare SUS foil without BLs and Sn only BL. The inset figures highlight the initial nucleation overpotential during Li plating (discharging). A voltage dip appeared at the initial stage of Li plating followed by a voltage plateau for the bare SUS foil. The gap between the voltage dip and plateau is defined as the overpotential for Li nucleation.^13^ A large overpotential, in general, indicates sluggish Li-metal nucleation and inhomogeneous Li-metal plating.^12,16,38^ To ensure the repeatability of the cell testing results for overpotentials and Coulombic efficiencies, 3 different individual cells for each sample were tested in this study. While a representative result of each sample is shown in Figure 2b, the values described here are the average values with error bars. The cell with the bare SUS foil exhibits a large overpotential of ∼30 ± 0.924 mV, indicating slow Li-metal nucleation. The SUS/Sn/C BL, SUS/C/Sn BL, and Sn only BL show overpotentials of 12 ± 0.924, 15 ± 0.924, and 8.33 ± 1.067 mV, respectively, which are much smaller than the bare SUS foil. In addition, the first-cycle Coulombic efficiencies were calculated from the voltage curves of SUS/Sn/C and SUS/C/Sn BL sputtered SUS foils, the bare SUS foil, and Sn only BL. The SUS/Sn/C BL and SUS/C/Sn BL exhibited a Coulombic efficiency of 87.6667 ± 0.533 and 86.3333 ± 0.533%, respectively, which are higher than the bare SUS foil (83.6667 ± 1.411%) but lower than Sn only BL (89.6667 ± 0.533%).

Figure 2b–e show the cycling performance of the SUS/Sn/C, SUS/C/Sn, Sn only BLs, and the bare SUS foil without BL, respectively. The bare SUS foil without BL was completely short-circuited in the eighth cycle as shown in Figure 2e. In sharp contrast, the SUS/Sn/C BL exhibited stable Li plating–stripping cycling performance without any short-circuit for 450 cycles (885 h) while the overall overpotential gradually increased during Li-plating (discharging), as shown in Figure 2b. The insets of Figure 2b show Li metal plating/stripping profiles at different cycling number ranges. At initial cycles, the nucleation overpotential of Li metal plating is negligible and the Li metal stripping profile shows a low voltage plateau. However, in the extended cycles, higher Li metal nucleation overpotential is observed. In addition, the stripping voltage profiles exhibit two distinct regions: (i) a low voltage plateau, followed by (ii) a sloped voltage curve at higher potential. The sloped voltage profile with a larger overpotential region (ii) is attributable to the contact loss between the solid electrolyte and Li metal-plated anode due to the void formation.^39,40^ Interestingly, we found that the Li metal stripping voltage does not always reach the cutoff voltage limit (1.0 V). We suspect that the fluctuations in the voltage profiles observed at the stripping side are due to the change in interfacial contact between Li metal-plated electrode and electrolyte caused during continuous plating and stripping cycles. During repeated Li metal plating and stripping cycles, isolated dead Li metals can form because of the void formation.^39,40^ When the isolated dead Li metals are reconnected by Li metal plating in a subsequent cycle, the dead Li metal can be stripped out. In this case, the stripping voltage profile does not reach the voltage cutoff but is limited by the stripping capacity. With the SUS/C/Sn BL, a short–short was observed in the 10th cycle, indicated by a sudden voltage drop to near-zero voltage (Figure 2c). For comparison, we evaluated the cycling stability of the Sn sputtered SUS foil without carbon. Figure 2d shows the Li-plating (discharging)/stripping (charging) cycling behavior of the Sn-deposited electrode. A short-circuit was observed in the 68th cycle when Sn only BL was used. These results demonstrate that the presence and position of the carbon layer play a pivotal role in stabilizing Li-plating (discharging)/stripping (charging) cycles and suppressing Li-dendrite growth, which will be discussed in the next section.

**Figure 3:**
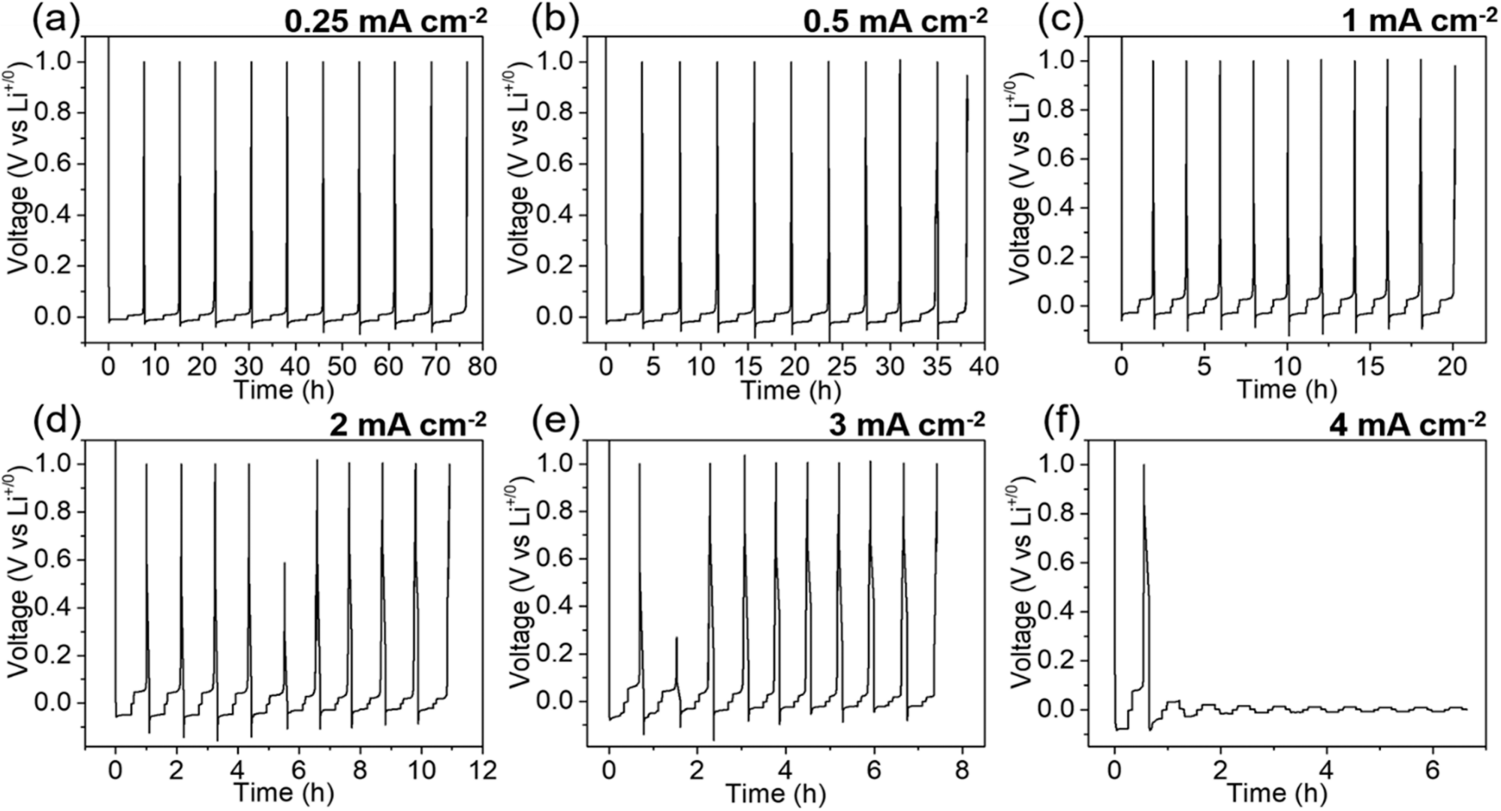
Galvanostatic charge–discharge profiles of SUS/Sn/C BL at (a) 0.25 mA cm^–2^, (b) 0.5 mA cm^–2^, (c) 1 mA cm^–2^, (d) 2 mA cm^–2^, (e) 3 mA cm^–2^, and (f) 4 mA cm^–2^ with a constant areal capacity of 1 mAh cm^–2^ to evaluate the critical current density.

As the SUS/Sn/C BL shows stable cycling performance, we further evaluated the critical current density (CCD) of the SUS/Sn/C BL. Figure 3 shows Li-plating (discharging)/stripping (charging) cycles at varied current densities from 0.25 to 4 mA cm^–2^ with a constant areal capacity of 1 mAh cm^–2^ for 10 cycles with a 5 min rest after each discharge and charge step. At current densities <2 mA cm^–2^, no noticeable short-circuit was observed for 10 cycles. At 3 mA cm^–2^, the voltage slightly decreases in the second cycle as shown in Figure 3e. However, the decreased voltage does not reach 0 V in sharp contrast to Figure 3f (4 mA cm^–2^) which was completely shorted. We expect the voltage drop in the second cycle at 3 mA cm^–2^ may originate from (i) a soft-short or (ii) an improved contact between Li metal-plated BL and solid electrolyte. Because Li metal plating/stripping cycle does not stop after the second cycle at 3 mA cm^–2^, we do not conclude the voltage drop is the complete short-circuit. In contrast, the cell was completely short-circuited after 2 cycles when a high current density of 4 mA cm^–2^ was applied.

### Li-Metal Plating Behaviors with SUS/Sn/C and SUS/C/Sn BLs

**Figure 4:**
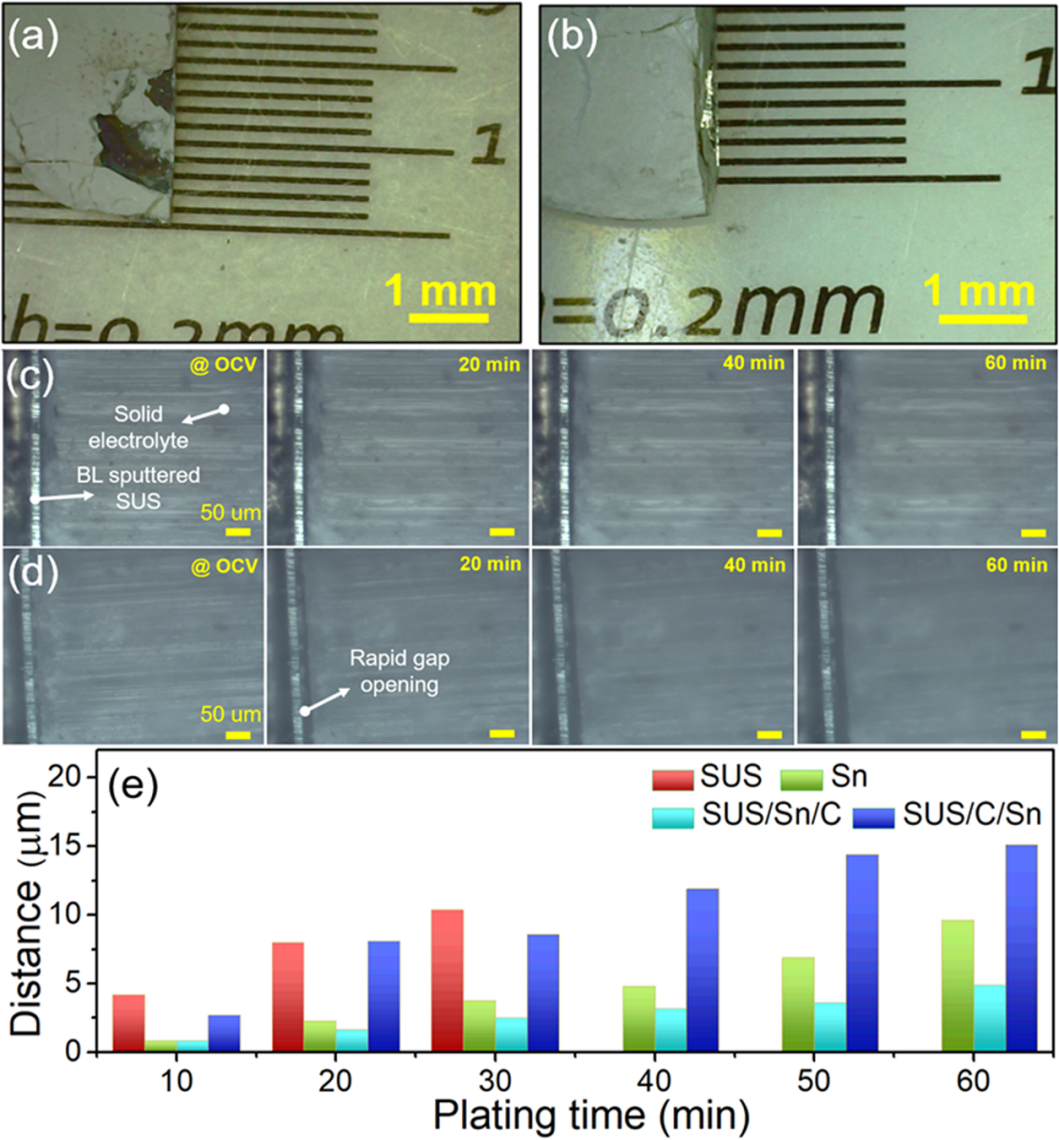
*Ex situ* digital optical microscopy images at the interface of the (a) solid electrolyte and SUS/Sn/C, and (b) soild electrolyte and SUS/C/Sn BLs after Li plating. (c) *In situ* optical microscopy images at the interface of the (c) solid electrolyte and SUS/Sn/C, and (d) solid electrolyte and SUS/C/Sn deposited SUS foil as a function of discharge time at 0.3 mA cm^–2^. (e) Distance between the solid electrolyte and the buffer layers (bare SUS foil, Sn, SUS/Sn/C and SUS/C/Sn) as a function of time estimated from the optical microscopy images.

To better understand the role of the position of the carbon layer in the BL in determining the Li-plating behavior, we employed *ex situ* digital optical microscopy and *in situ* cross-sectional optical microscopy. Figure 4a,4b present the *exsitu* optical microscopy images (top-view) of the SUS/Sn/C and SUS/C/Sn BLs after Li plating at 1 mA cm^–2^ for 15 h (areal capacity: 15 mAh cm^–2^). Their Li-plating (discharging) profiles are shown in Figure S2. Notably, these cells were not short-circuited during Li plating for 15 h. The cells were disassembled and the Li metal counter electrode was carefully removed from the pellet using a blade inside Ar-filled glovebox. Subsequently, the pellets, consisting of solid electrolyte |Li-plated BL| SUS foil, were cut in half using a blade inside Ar-filled glovebox. The top layer of the solid electrolyte was carefully removed to observe the interface between the solid electrolyte and BL using a digital optical microscope. All the sample preparation steps and optical microscopy measurements were conducted inside an Ar-filled glovebox to avoid any potential contamination from air exposure. As shown in Figure 4a, the carbon layer with black color was observed between the solid electrolyte and plated Li metal for the case of SUS/Sn/C BL. We could not observe any shiny Li metals at the interface between the solid electrolyte and BL. SEM/EDS analysis also confirmed that the black films observed in Figure 4a are the carbon layer (Figure S3). Importantly, we could not observe any noticeable oxygen signal from the area where we conducted EDS mapping in Figure S3. The absence of an oxygen signal indicates that the material underneath the LPSCl solid electrolyte is the carbon layer, instead of other potential decomposition products such as lithium carbonate. When we scratched the carbon layer, Li metal (a shiny metallic piece) was observed, demonstrating that Li metal was plated between the carbon BL and SUS foil. The cross-sectional optical microscopy image in Figure S4 also demonstrates the Li-metal plating underneath the carbon layer. In contrast, Li metal was observed when the solid electrolyte layer was removed for the SUS/C/Sn BL, as shown in Figure 4b. This observation likely demonstrates that the Li metal was plated between the solid electrolyte and BL. We expect the Li-metal plating beneath the carbon layer to suppress the detrimental decomposition of the solid electrolyte by avoiding direct contact between the Li metal and solid electrolyte in the SUS/Sn/C deposited BL. In addition, the presence of the carbon layer on top of the plated Li metal could act as a physical barrier to suppress Li dendrite growth and penetration into the solid electrolyte, which will be discussed further in a later section.

We employed *in situ* cross-sectional optical microscopy analysis to understand how the BL configurations (SUS/Sn/C vs SUS/C/Sn) affect the uniformity of Li metal plating on the SUS foil current collector. The customized *in situ* solid-state battery cell design is shown in Figure S5. When a fixed current rate is applied, how rapidly the gap between the solid electrolyte and SUS foil opens is inversely proportional to the homogeneity of Li metal plating (Figure S6). When Li metal plating is homogeneous on the SUS foil, the gap opening should be gradual and slow. In contrast, the uneven Li metal plating will open the gap between the solid electrolyte and SUS foil more significantly as shown in Figure S6. Figure 4c,d present *in situ* cross-sectional optical microscopy images of the area between the solid electrolyte and SUS foil with SUS/Sn/C and SUS/C/Sn BLs, respectively, as a function of Li-plating (discharging) time at 0.3 mA cm^–2^. The capacity resulting from the Li–Sn alloying reaction is 0.053 mAh cm^–2^ based on the mass of Sn on the BL, which is only ∼17% of the total capacity. Therefore, the majority of the capacity obtained from the in situ cross-sectional optical microscopy analysis has to be related to Li plating. The voltage profiles of the *in situ* cross-sectional optical microscopy experiments are shown in Figure S7. The higher overpotential observed in these *in situ* experiments is attributable to the higher cell resistances in the specific setup of the *in situ* solid-state battery cells with a relatively low stacking pressure (<1 MPa). The recorded videos can be found in Supporting Videos S1–S2. Before cycling, the gap between the SUS foil and the solid electrolyte for the SUS/Sn/C BL is smaller than that for the SUS/C/Sn BL, which indicates that the contact between the SUS/Sn/C BL deposited SUS foil and the solid electrolyte is better than that between the SUS/C/Sn BL deposited SUS foil and the solid electrolyte. During the Li plating (discharging), the gap between the SUS/Sn/C BL deposited SUS foil and the solid electrolyte gradually increases (Figure 4c). In contrast, for the SUS/C/Sn BL deposited SUS foil (Figure 4d), the gap opening between the SUS foil and solid electrolyte is much greater and faster than the SUS/Sn/C BL case during Li plating (discharging).

For comparisons, Sn only BL and bare SUS foil without BL were also tested with *in situ* cross-sectional optical microscopy measurements (Figure S8 and Supporting Videos S3–S4). In the case of Sn only BL, a gradual gap opening between the solid electrolyte and Sn deposited SUS foil was found, similar to the SUS/Sn/C BL system. However, the bare SUS foil exhibited a rapid gap opening between the solid electrolyte and SUS foil. In addition, a crack in the solid electrolyte started evolving after 20 min of Li metal plating and the cell was short-circuited after 30 min of Li metal plating.

Figure 4e summarizes the gap opening between the solid electrolyte and SUS foil as a function of Li plating time with different BLs. The SUS/Sn/C BL shows the slowest and gradual gap opening between the solid electrolyte and SUS foil. In contrast, SUS/C/Sn BL exhibits a much faster gap opening than the SUS/Sn/C BL. Interestingly, Sn only BL shows a slower gap opening than the SUS/C/Sn BL. This behavior could be attributable to poor adhesion between the sputtered carbon and SUS foil. The SUS/C/Sn BL was easily detached from the SUS foil, as shown in Figure S9. The poor contact between BL and SUS foil can induce uneven Li plating. As expected, the bare SUS foil exhibits the fastest gap opening. Because the bare SUS foil was short-circuited after 30 min of discharging, the gap opening distance for the bare SUS foil was not plotted after 30 min in Figure 4e. The speed of the gap opening shows the trend of the bare SUS (fastest) > SUS/C/Sn > Sn only > SUS/Sn/C (slowest). Intriguingly, this trend is inversely proportional to the cycling performance. The slower the gap opening is, the longer it cycles without short-circuiting (Figure 2). As we discussed above, a rapid gap opening between SUS foil and solid electrolyte indicates inhomogeneous Li metal plating. Therefore, we attribute the stable cycling performance of the SUS/Sn/C BL to their ability to homogenize Li metal plating.

### Li-Metal Plating and Stripping Mechanisms with the SUS/Sn/C BL

**Figure 5:**
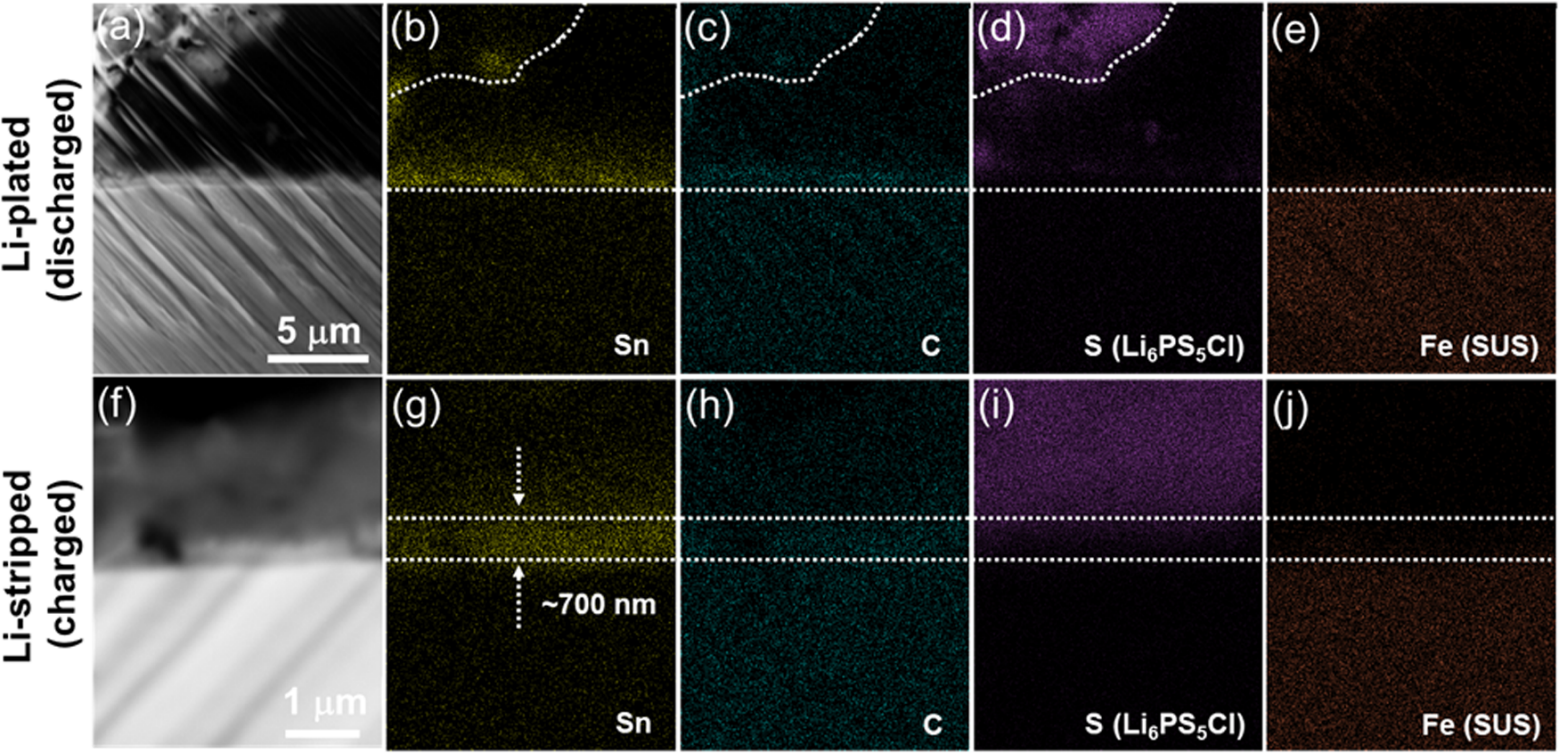
(a) SEM image and corresponding EDS mapping of (b) Sn, (c) C, (d) S (for Li_6_PS_5_Cl solid electrolyte), and (e) Fe (SUS) of the discharged SUS/Sn/C BL. (f) SEM image and corresponding EDS mapping of (g) Sn, (h) C, (i) S (for Li_6_PS_5_Cl solid electrolyte), and (j) Fe (SUS) of the charged SUS/Sn/C BL.

To understand Li-metal plating and stripping behaviors with the SUS/Sn/C BL, we used cryo-focused ion beam (cryo-FIB) and SEM/EDS analysis after Li-metal plating and stripping at 1 mA cm^–2^. We used cryo-FIB for the sample preparation to prevent Li-metal melting and potential decomposition of interphase materials due to the local heating during the FIB process. Because sulfide solid-electrolyte and interphase materials are air- and moisture-sensitive,^41,42^ an air-free sample preparation and transfer system was implemented to protect the samples from contamination. Figure 5a–e show the SEM image and corresponding EDS mapping results of the SUS/Sn/C BL deposited SUS foil after Li-metal plating at 1 mA cm^–2^ with an areal capacity of 2 mAh cm^–2^. After Li-metal plating, a dark area appeared in Figure 5a with no S signal (representing Li_6_PS_5_Cl solid electrolyte) and no Sn signal in the EDS mapping between the solid electrolyte and SUS foil. We expect that this dark area corresponds to Li metal plated after discharging. Interestingly, the Sn signal shows a gradient from the SUS foil (high intensity) to the Li_6_PS_5_Cl solid electrolyte (low intensity). This gradient demonstrates that most of the Sn metal remained on the SUS foil after Li metal plating. The phase separation of Li metal and Sn-rich phase is attributable to the fact that the solid-solution alloy reaction between Li metal and Sn metal is limited to Li_22_Sn_5_ in contrast to Ag or Au that can form a wide range of solid-solution up to very high Li concentrations.^24,38^ The formation of Li metal and the phase separation of Li and Sn were also evidenced by *ex situ* XRD analysis, confirming the coexistence of Li metal and Li–Sn alloy (Li_22_Sn_5_), as shown in Figure S10. While a relatively concentrated carbon signal was detected on the surface of the SUS foil, it is difficult to conclude where the carbon layer presents because (i) the carbon signal is weak and (ii) the weak carbon signal is also found at the interface between the Li metal plated and the solid electrolyte. This makes it difficult to confirm whether the carbon layer is present on the top of the Li metal. However, the optical microscopy image in Figure 4a clearly demonstrates the presence of the carbon layer on top of the Li metal. Figure 5f–j show SEM images of the interface between the SUS/Sn/C deposited BL and Li_6_PS_5_Cl solid electrolyte after Li stripping at 1 mA cm^–2^ up to 1.0 V (vs Li/Li^+^). The thickness of the SUS/Sn/C BL shortened significantly to ∼700 nm after Li stripping. This result indicates that the plated Li metal was removed from the anode during the charging process. The *ex situ* XRD analysis also confirmed the disappearance of Li metal and Li_22_Sn_5_ phases and the recovery of Sn-metal peaks after charging (Figure S10). Notably, the Sn and carbon layer remained at the interface between the solid electrolyte and SUS foil current collector. Although the carbon signal is weak and it is difficult to confirm the thickness in Figure 5h, the summed line scan of the carbon signal in Figure S11 clearly demonstrates the presence of a thin carbon layer between the solid electrolyte and SUS foil. In addition, Sn and C coexist in the same position. It is likely that the Sn and C were mixed after a Li-plating (discharging)/stripping (charging) cycle.

## Discussion

**Figure 6:**
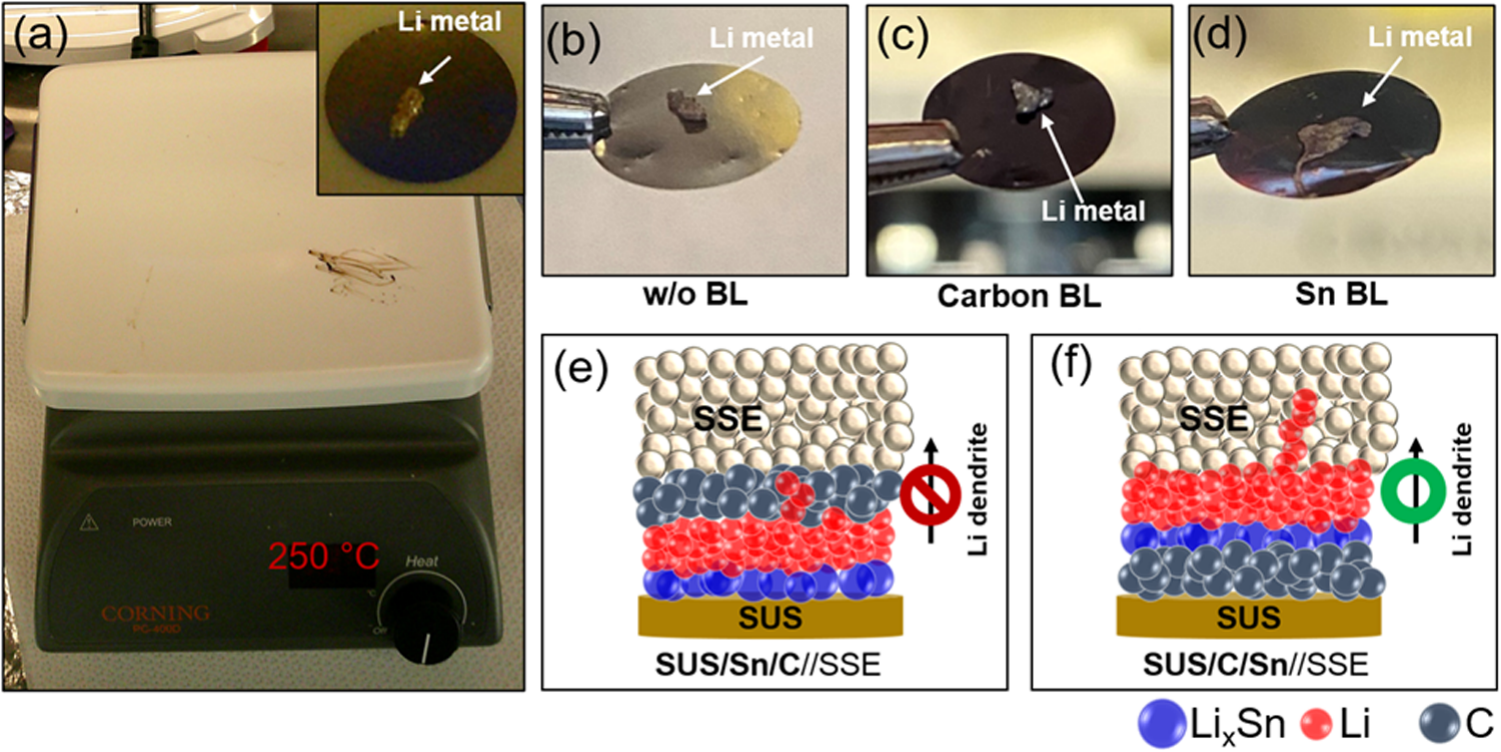
(a) Experimental setup for lithiophobicity evaluation. Digital photo images of melted Li metal on SUS foils (b) without BL, (c) with carbon BL, (d) with Sn BL. Schematics of Li metal growth behaviors with (e) SUS/Sn/C BL and (f) SUS/C/Sn BL.

Our study demonstrates that the dual SUS/Sn/C BL results in improved Li-plating (discharging)/stripping (charging) cycling without noticeable short-circuit compared to Sn only and the dual SUS/C/Sn BLs (Figure 2). This improvement can be explained by the presence of the protective carbon layer on top of the Li metal, as demonstrated in Figure 4a. The carbon layer may (i) prevent direct contact between the sulfide solid electrolyte and Li metal and (ii) serve as a physical barrier for Li-dendrite penetration. To better understand how the carbon layer prevents dendritic Li metal growth toward the solid electrolyte, we evaluated the lithiophobicity of sputtered carbon and Sn on SUS foils. Figure 6a shows an experimental setup to evaluate the wettability of Li metal on SUS foil without BL, carbon BL, and Sn BL sputtered SUS foils. A small piece of Li metal (∼1 mg) was placed on the carbon or Sn sputtered SUS foils and the bare SUS foil without BL, followed by heat treatment on a hot plate (temperature setup: 250 °C) inside Ar-filled glovebox (Inset of Figure 6a: before heat treatment). Figure 6b–d show digital photo images of melted Li metal on SUS foil without BL, carbon BL, and Sn BL sputtered SUS foils, respectively. Similar to the bare SUS foil without BL (Figure 6b), melted Li metal did not show a good wettability on carbon sputtered SUS foil (Figure 6c), indicating its lithiophobic nature. In contrast, Li metal was well spread on the Sn sputtered SUS foil, as illustrated in Figure 6d, demonstrating its lithiophilic property. We expect that Li metal prefers to grow toward the SUS foil side where lithiophilic Sn metal is deposited rather than the solid electrolyte side where the lithiophobic carbon layer presents in the SUS/Sn/C BL system (Figure 6e). On the contrary, the carbon layer on the SUS foil will function as a current collector and does not prevent Li dendrite growth toward the solid electrolyte in the SUS/C/Sn BL system (Figure 6f). This finding highlights the importance of the lithiophobic carbon layer in BL development in suppressing Li dendrite growth and penetration toward the solid electrolyte. In particular, the lithiophobic carbon layer at the solid electrolyte side is pivotal in preventing Li dendrite growth.

## Conclusions

In this study, we demonstrated a potential dual BL consisting of Sn and C deposited on a current collector. The SUS/Sn/C dual layer exhibited stable Li-plating (discharging)/stripping (charging) cycling without any noticeable short-circuit compared to SUS/C/Sn dual BL and Sn-only BL. The uniform Li-metal plating in the SUS/Sn/C dual layer at the interface of carbon and the current collector is attributed to the homogeneous distribution of lithophilic Sn on the current collector. The carbon layer on top of the Sn nanoparticles may serve as a physical barrier to prevent Li-dendrite penetration of the solid electrolyte due to its lithiophobic nature. In contrast, when SUS/C/Sn dual BL was applied, the Li metal was plated at the interface between the solid electrolyte and the BL, which cannot avoid direct contact between the Li metal and solid electrolyte nor suppress Li dendrite growth toward the solid electrolyte. This study demonstrates the importance of the carbon position in the BL in suppressing Li-metal penetration of the solid electrolyte which challenges the safety and longevity of SSBs.

## Methods

### Material Synthesis

The dual buffer layers were deposited using direct current (DC) magnetron sputtering (MSE supplies, MA0600) inside an Ar-filled glovebox. The pressure was maintained at 5 Pa during the deposition in the vacuum chamber, and the power was maintained at 10 W. Metallic tin (Sn), and carbon targets with a purity of 99.99% and a diameter of 50 mm were used as sputtering targets. The sputtering was kept constant at 5 min for Sn and 20 min in the case of carbon to achieve a uniform distribution.

### Characterization

A Rigaku Miniflex 600 high-resolution diffractometer equipped with a Cu–Kα source (λ = 0.15418 nm) was used for recording the XRD patterns of the metal-deposited SUS foils and SUS/Sn/C BL deposited SUS foil after Li plating and Li stripping. Microstructural analysis of the samples was conducted using a FEI Quanta FEG-250 and Phenom PW-100–017 scanning electron microscope. EDS elemental mappings were recorded using a Bruker Quantax EDS detector. *Ex situ* digital images of the lithiated BLs were recorded using a digital optical microscope (7″ LCD Digital Microscope 1200X, 12MP 1080P, Dcorn) inside the Ar-filled glovebox. *In situ* optical microscopy study was conducted using a quartz cell with transparent windows and a rectangular-shaped empty space inside as the cell housing, in which a sandwich structure of lithium metal, the separator, and the BL-coated SUS foil was assembled. The fabricated cell was placed on the stage of an optical microscope (MM500T microscope, MTI) with a transparent window facing the lens. A Scios 2 Dual Beam cryo-FIB/SEM equipped with an Octane Elite Super EDS detector was used for recording the EDS images for the charged and discharged buffer layers. The samples were transferred to the microscope using an air-free Leica EM VCT500 cryo-transfer system. The cryo-SEM and cryo-FIB experiments were performed on a Scios 2 Dual Beam SEM/FIB equipped with a Leica VCT cryogenic stage cooled to −150 °C. The samples were prepared in an Ar-filled glovebox and then coated with 7 nm of Pt using a cryo-sputter coater (ACE 600) before being transferred to the cryo-SEM/FIB under high vacuum using the Leica VCT500 shuttle. EDS maps were obtained using an EDAX Octane Elite detector.

### Electrochemical Testing

To investigate the electrochemical performance, half cells were constructed using our customized cells. The customized cells consisted of a 6.35 mm poly(etheretherketone) (PEEK) die and two steel rods. The customized cells were placed between two steel plates, and a stack pressure was applied between them. In this assembly, 50 mg of argyrodite-type lithium phosphorus sulfur chloride (Li_6_PS_5_Cl, *D*_50_ ∼ 1 μm, Ampcera Inc.) solid electrolyte was loaded into the PEEK die and pressed between the two rods at 300 MPa for 15 min to form a 1 mm thick pellet. BL-deposited SUS foil was pressed on one side of the solid-electrolyte pellet at 300 MPa to ensure good contact between the BL and solid electrolyte. Li metal was placed on the other side of the solid-electrolyte pellet and pressed at 20 MPa for 15 min. A stack pressure of ∼5 MPa was applied during the cell operation. Electrochemical tests of the BL-deposited electrodes were conducted at a current density of 1 mA cm^–2^ and a fixed areal capacity of 1 mAh cm^–2^. The cells were rested for 6 h at OCV before cycling and limited by voltage (−1,1) during the discharge/charge cycles. All the electrochemical testing was conducted using a Maccor Model 2200 cycler at 50 °C. To confirm the repeatability, we tested 3 different individual cells for each sample.

## Supporting Information Available

XRD and SEM/EDS for Sn deposited BL. Li plating profiles of SUS/Sn/C and SUS/C/Sn BLs. SEM/EDS analysis of Li metal plated SUS/Sn/C BL. Digital optical microscopy image of Li metal plated SUS/Sn/C BL (Cross Section). A photo image of the customized in situ solid-state battery cell. A schematic of the Li metal distribution of nonuniform vs homogeneous Li metal plating. Discharge profiles of in situ cells. In situ optical microscopy images at the interface of the solid electrolyte and SUS foil with Sn only BL and without any BLs. SEM image of SUS/C/Sn BL. XRD patterns of pristine, discharged and discharged–charged SUS/Sn/C BL cycled at 1 mA cm^–2^ current density. SEM image, carbon mapping, and corresponding summed line scan of the carbon signal (PDF)
Sn-C (Video S1) (MP4)
C-Sn (Video S2) (MP4)
Sn (Video S3) (MP4)
SUS (Video S4) (MP4)
