# Supplementary material for: Tin–Carbon Dual Buffer Layer to Suppress Lithium Dendrite Growth in All-Solid-State Batteries
Source: ACS Nano. 2025 Apr 29;19(18):17347–56. doi: 10.1021/acsnano.4c16271 (PMC12080328; doi:10.1021/acsnano.4c16271)
Supplement: Supplementary file 1 — nn4c16271_si_001.pdf [file nn4c16271_si_001.pdf]

# Tin–Carbon Dual Buffer Layer to Suppress Lithium Dendrite Growth in All-Solid-State Batteries

*Venkata Sai Avvaru,*[a] *Tofunmi Ogunfunmi,*[b,c] *Seonghun Jeong,*[a] *Mouhamad Said Diallo,*[a,b] *John Watt,*[d] *Mary C. Scott,*[b,c] *Haegyeom Kim*[a,*]

a. Materials Sciences Division, Lawrence Berkeley National Laboratory, Berkeley, CA, 94720 USA
b. Department of Materials Science and Engineering, University of California, Berkeley, CA, 94720, USA
c. Molecular Foundry Division, Lawrence Berkeley National Laboratory, Berkeley, CA 94720, USA
d. Center for Integrated Nanotechnologies, Los Alamos National Laboratory, Los Alamos, New Mexico 87545, USA

Corresponding author: H. K. (haegyumkim@lbl.gov)

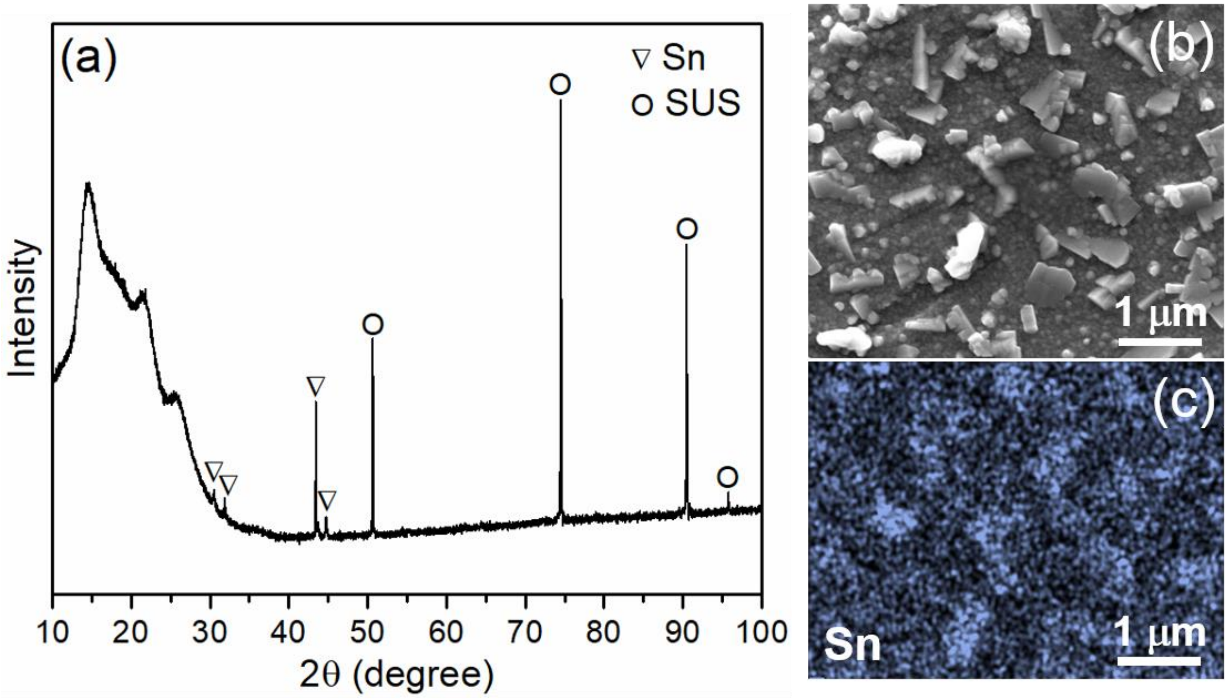


**Figure S1.** (a) XRD analysis of the Sn deposited on the SUS foil. (b) SEM images of Sn metal nanoparticles and corresponding EDS mapping of the Sn deposited SUS foil.

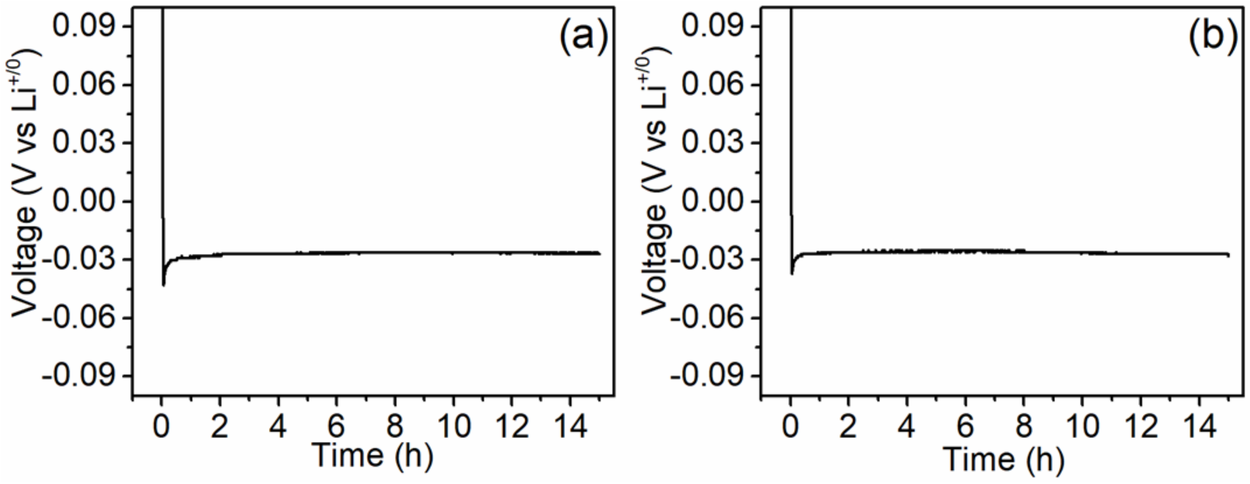


**Figure S2.** Discharge profile of (a) SUS/Sn/C and (b) SUS/C/Sn at a current density of 1 mA cm$^{-2}$ for 15 h.

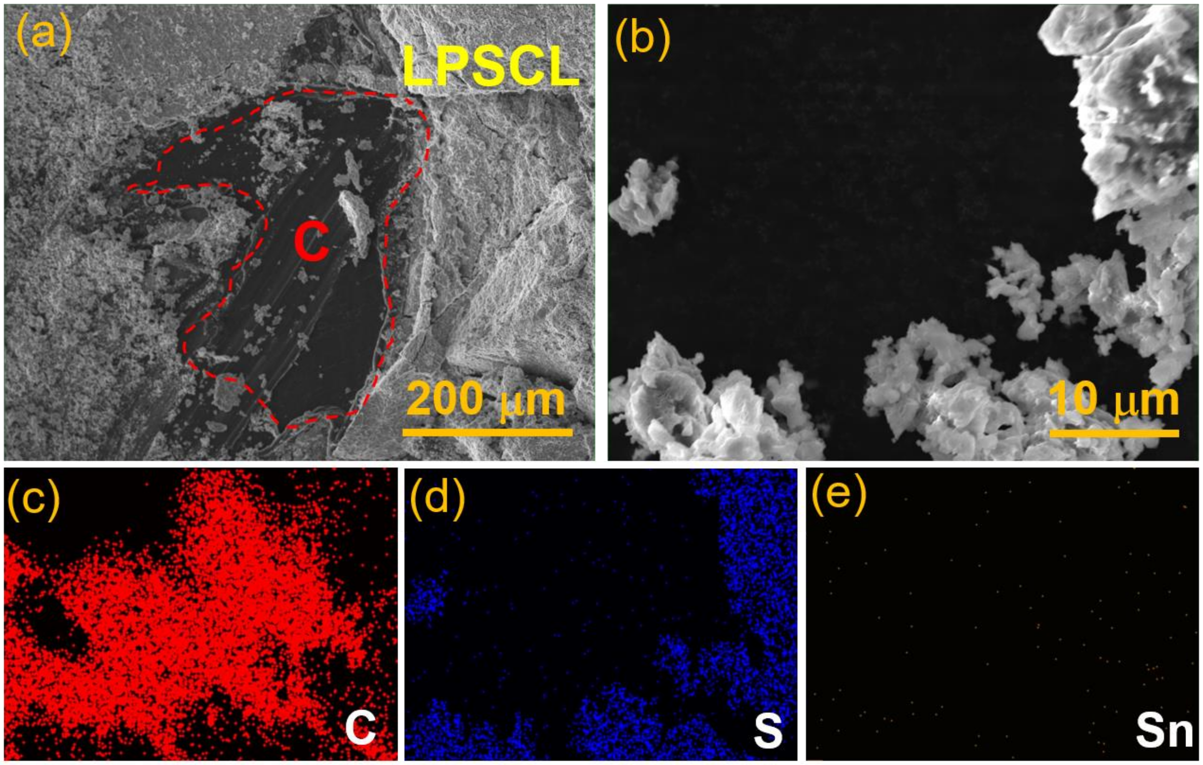


**Figure S3.** (a) SEM image of the Li metal-plated SUS/Sn/C BL. (b) SEM image and corresponding EDS mapping of (c) C, (d) S (for $Li_6PS_5Cl$ solid electrolyte), and (e) Sn of the Li metal-plated SUS/Sn/C BL.

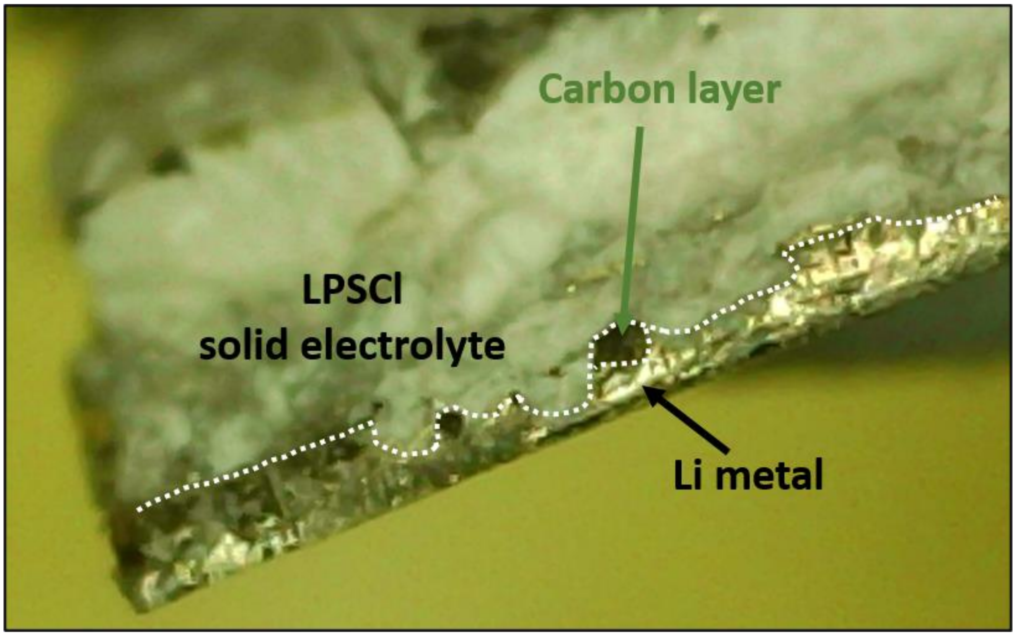


**Figure S4.** Digital optical microscopy image of cycled SUS/Sn/C (cross section).

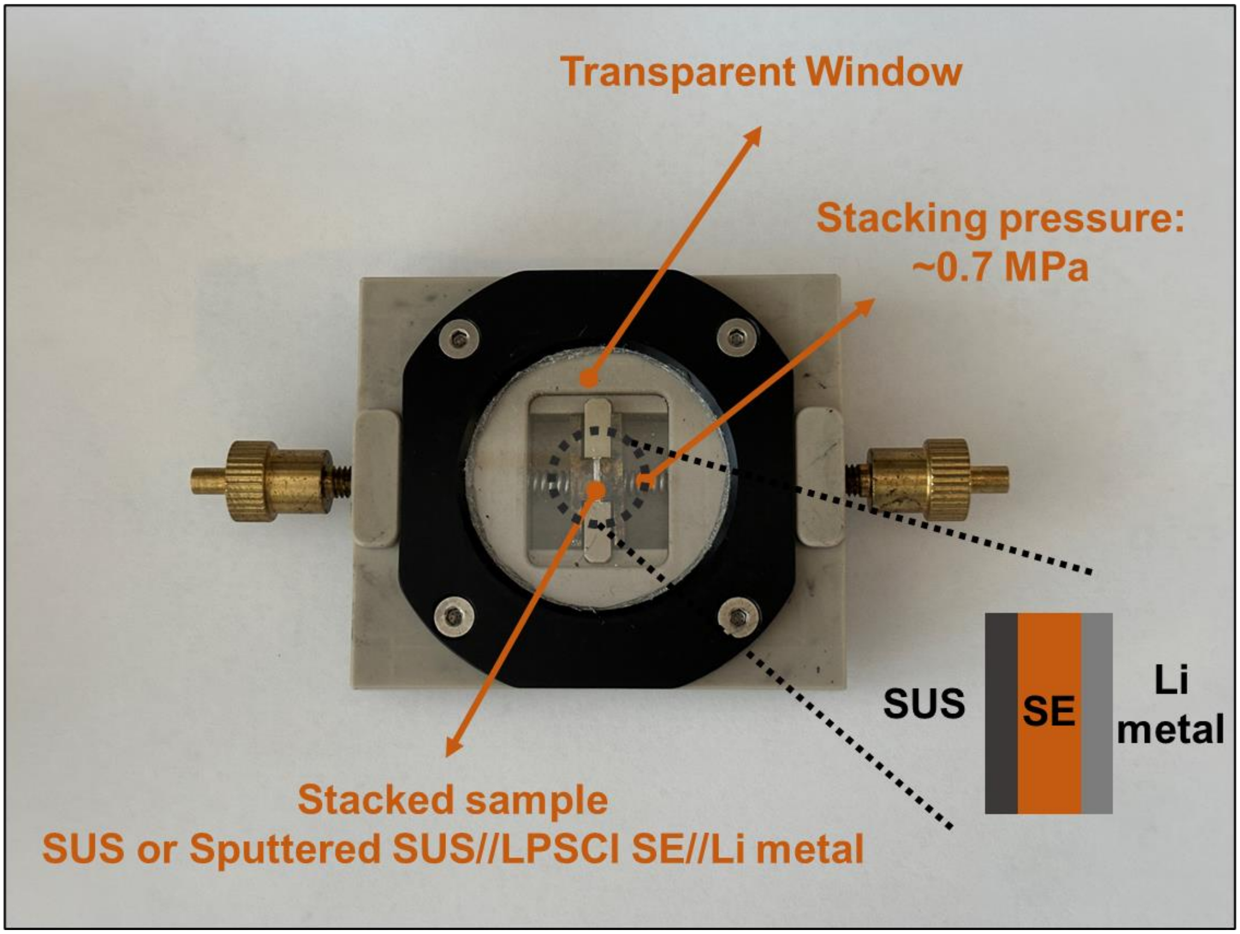


**Figure S5.** Customized i*n-situ* solid-state battery cell.

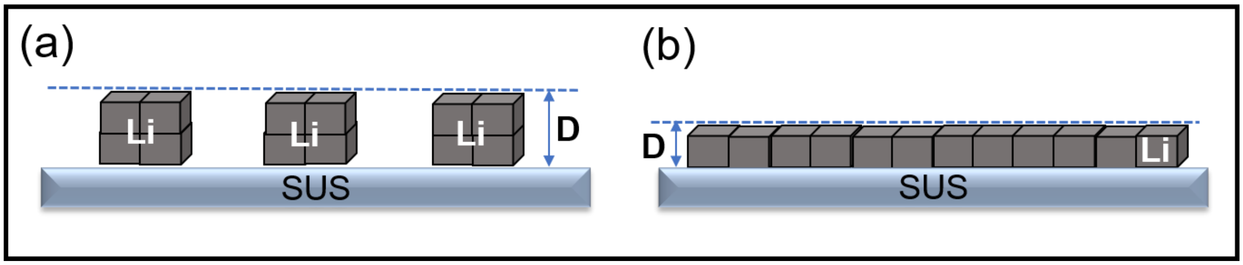


**Figure S6.** Schematic representation of the Li metal distribution. (a) non-uniform Li plating and (b) homogenous Li plating.

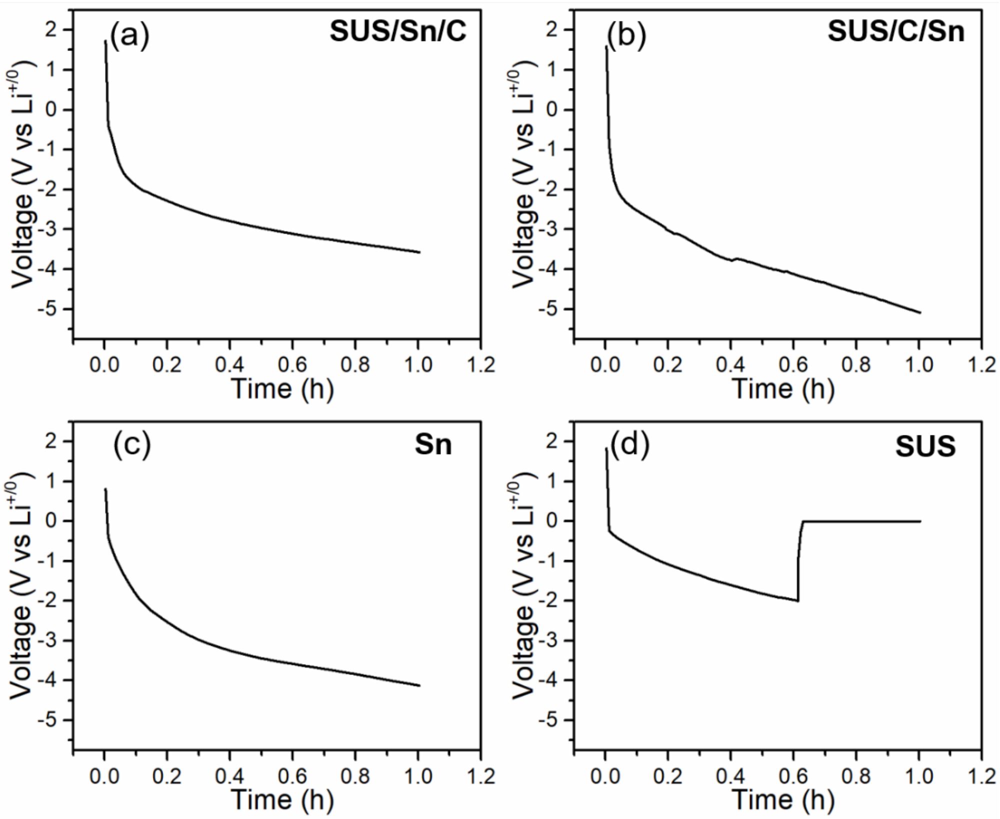


**Figure S7.** Discharge profile of (a) SUS/Sn/C, (b) SUS/C/Sn, (c) Sn only and (d) bare SUS foil at a current density of 0.3 mA $cm^{-2}$.

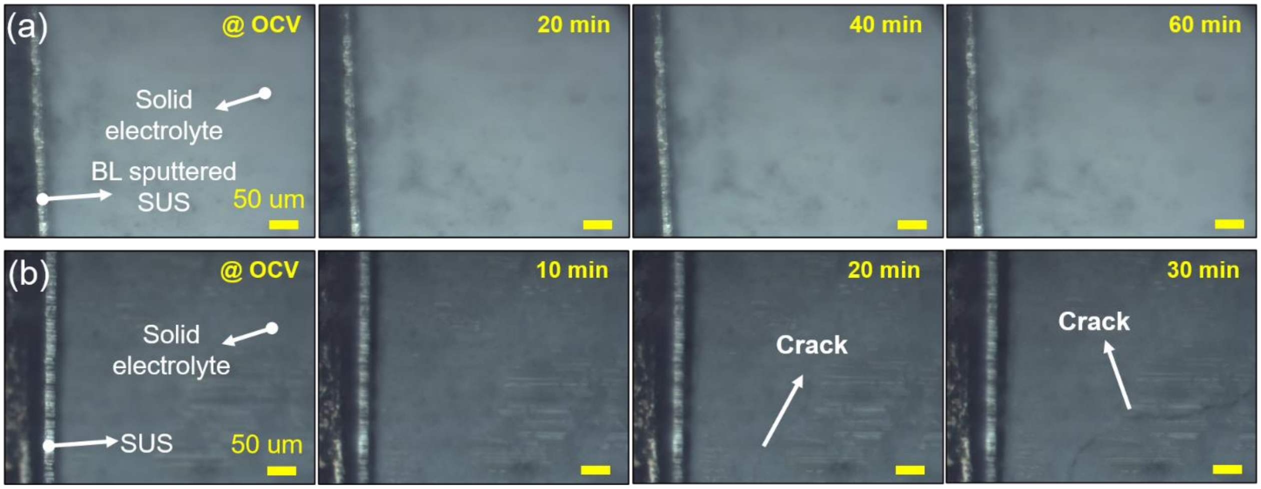


**Figure S8.** *In-situ* optical microscopy images at the interface of the solid electrolyte and SUS foil as a function of discharge time at 0.3mA $cm^{-2}$ with (a) Sn only BL and (b) bare SUS foil.

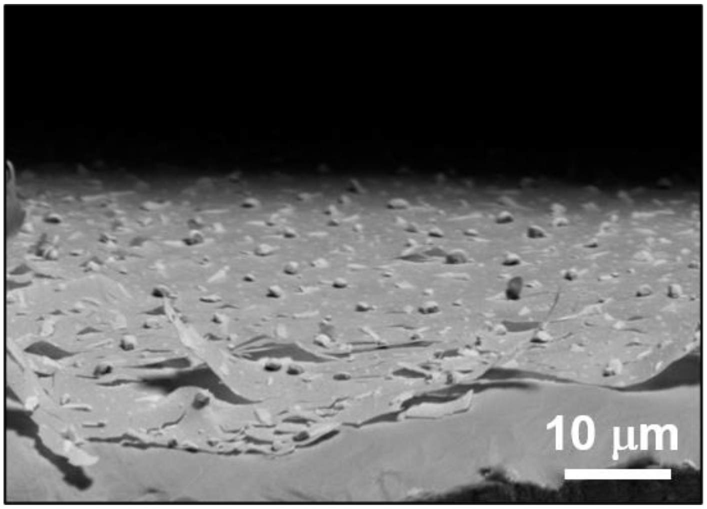


**Figure S9.** SEM image of SUS/C/Sn BL.

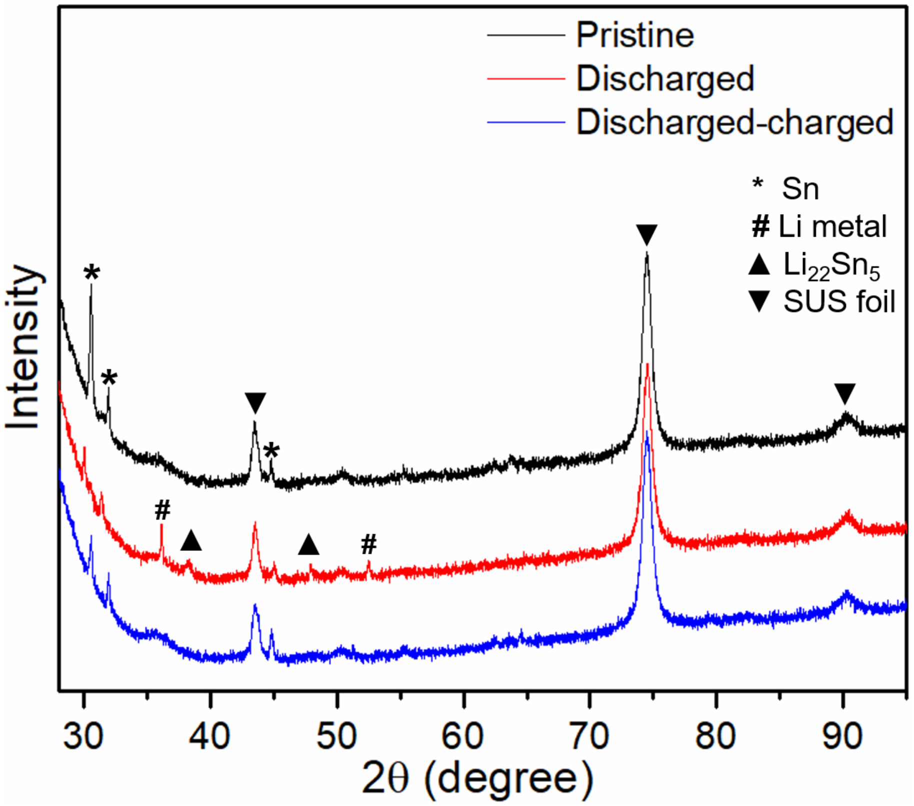


**Figure S10.** XRD patterns of pristine, discharged and discharged-charged SUS/Sn/C BL cycled at 1 mA $cm^{-2}$ current density.

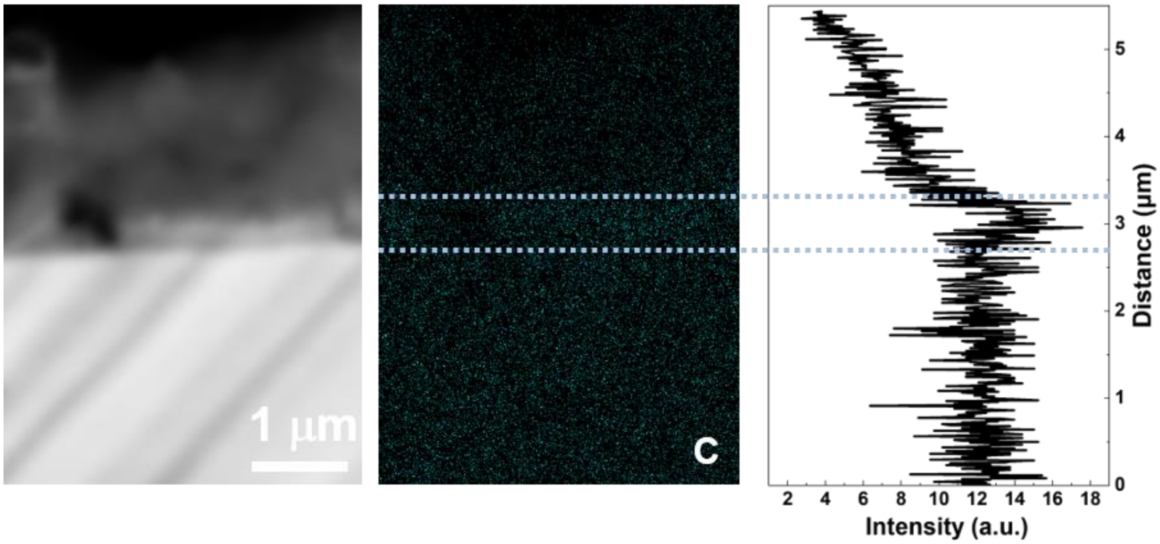


**Figure S11.** SEM image, carbon mapping, and corresponding summed line scan of the carbon signal.

**Videos for In-situ Optical microscopy analysis are shown in Videos S1-S4.**

**Video S1:** SUS/Sn/C BL

**Video S2:** SUS/C/Sn BL

**Video S3:** SUS/Sn BL

**Video S4:** SUS only
